# A Novel Interplay between Rap1 and PKA Regulates Induction of Angiogenesis in Prostate Cancer

**DOI:** 10.1371/journal.pone.0049893

**Published:** 2012-11-15

**Authors:** Jyotsana Menon, Robert C. Doebele, Suzana Gomes, Elena Bevilacqua, Katie M. Reindl, Marsha Rich Rosner

**Affiliations:** 1 Ben May Department for Cancer Research, The University of Chicago, Chicago, Illinois, United States of America; 2 School of Medicine, Division of Medical Oncology, University of Colorado Anschutz Medical Campus, Aurora, Colorado, United States of America; 3 Department of Biological Sciences, North Dakota State University, Fargo, North Dakota, United States of America; Rush University Medical Center, United States of America

## Abstract

Angiogenesis inhibition is an important therapeutic strategy for advanced stage prostate cancer. Previous work from our laboratory showed that sustained stimulation of Rap1 by 8-pCPT-2'-O-Me-cAMP (8CPT) via activation of Epac, a Rap1 GEF, or by expression of a constitutively active Rap1 mutant (cRap1) suppresses endothelial cell chemotaxis and subsequent angiogenesis. When we tested this model in the context of a prostate tumor xenograft, we found that 8CPT had no significant effect on prostate tumor growth alone. However, in cells harboring cRap1, 8CPT dramatically inhibited not only prostate tumor growth but also VEGF expression and angiogenesis within the tumor microenvironment. Subsequent analysis of the mechanism revealed that, in prostate tumor epithelial cells, 8CPT acted via stimulation of PKA rather than Epac/Rap1. PKA antagonizes Rap1 and hypoxic induction of 1α protein expression, VEGF production and, ultimately, angiogenesis. Together these findings provide evidence for a novel interplay between Rap1, Epac, and PKA that regulates tumor-stromal induction of angiogenesis.

## Introduction

Prostate cancer is the second leading cause of cancer-related death in men in the United States [1]. The high morbidity and mortality associated with the onset of hormone-refractory, metastatic prostate cancer mandates the need for innovative treatment regimens to improve the prognosis of this disease. Several strategies have been used to target angiogenesis in prostate cancer including blockade of pro-angiogenic factors like vascular endothelial growth factor (VEGF) via monoclonal antibodies or small molecule inhibitors targeting downstream signaling effector pathways like the VEGF receptor tyrosine kinase pathway [2], [3], [4]. However, the major shortcoming to this approach is the significant number of pro-angiogenic factors, apart from VEGF, that can induce angiogenesis and thereby evade these agents. An alternative to inhibiting one or more of the pro-angiogenic factors is to identify signaling molecules that function to regulate angiogenesis [5].

Several studies have implicated Rap1 as a mediator of angiogenesis [5], [6], [7], [8], [9], [10], [11]. Defective angiogenesis and hematopoiesis were observed in mice lacking Rap1a or Rap1b, and a mechanism involving integrins and VEGF receptors in endothelial cells was recently reported [12], [13], [14], [15], [16], [17], [18]. Thus, loss of Rap1 inhibits angiogenesis during development, consistent with the role Rap1 plays in cell signaling, integrin-mediated cell adhesion and cell-cell junctions, functions that are important for tubular structure formation. The cAMP derivative 8CPT-2Me-cAMP (8CPT) is a potent agonist of Epac, a guanine nucleotide exchange factor for Rap1, and a weak agonist of PKA [18], [19]. Previous work from our laboratory showed that, in primary human microvascular endothelial cells, prolonged stimulation of Rap1 either by Epac activation following 8CPT treatment or by expression of constitutively activated Rap1 A63E (cRap1) inhibits chemotaxis and angiogenesis [8], [9]. Thus, angiogenesis can also be inhibited in endothelial cells, when Rap1 is subject to prolonged stimulation by drugs or mutation. Together these studies suggest that the degree of Rap1 activation is critical to the angiogenic process.

PKA has also been linked to angiogenesis as both a positive and negative regulator [20], [21], [22], [23], [24], [25], [26]. Increased activity of PKA promotes endothelial tube formation, thus promoting angiogenesis [27]. Conversely, PKA activation causes the phosphorylation of the transcriptional repressor Id1 [28] and disrupts its nucleo-cytoplasmic shuttling, thus inhibiting angiogenesis [29]. In addition, either over-expression of the catalytic subunit of PKA or pharmacological activation of PKA induces the death of endothelial cells by apoptosis, suppressing angiogenesis [22], [23]. Altogether these results indicate that angiogenesis results from a balance of pro-and anti-angiogenic factors including Rap1 and PKA, and either extreme will have deleterious effects.

The aim of this study was to determine whether Rap1 regulates angiogenesis in prostate tumors. Our results suggest that constitutive Rap1 activation in human prostate tumor cells promotes hypoxic induction of VEGF and angiogenesis, and PKA antagonizes this effect. Furthermore, our studies suggest that 8CPT treatment can inhibit angiogenesis via two different mechanisms involving PKA activation in prostate tumor cells, as shown here, and Epac/Rap1 activation in endothelial cells [8].

**Figure 1:**
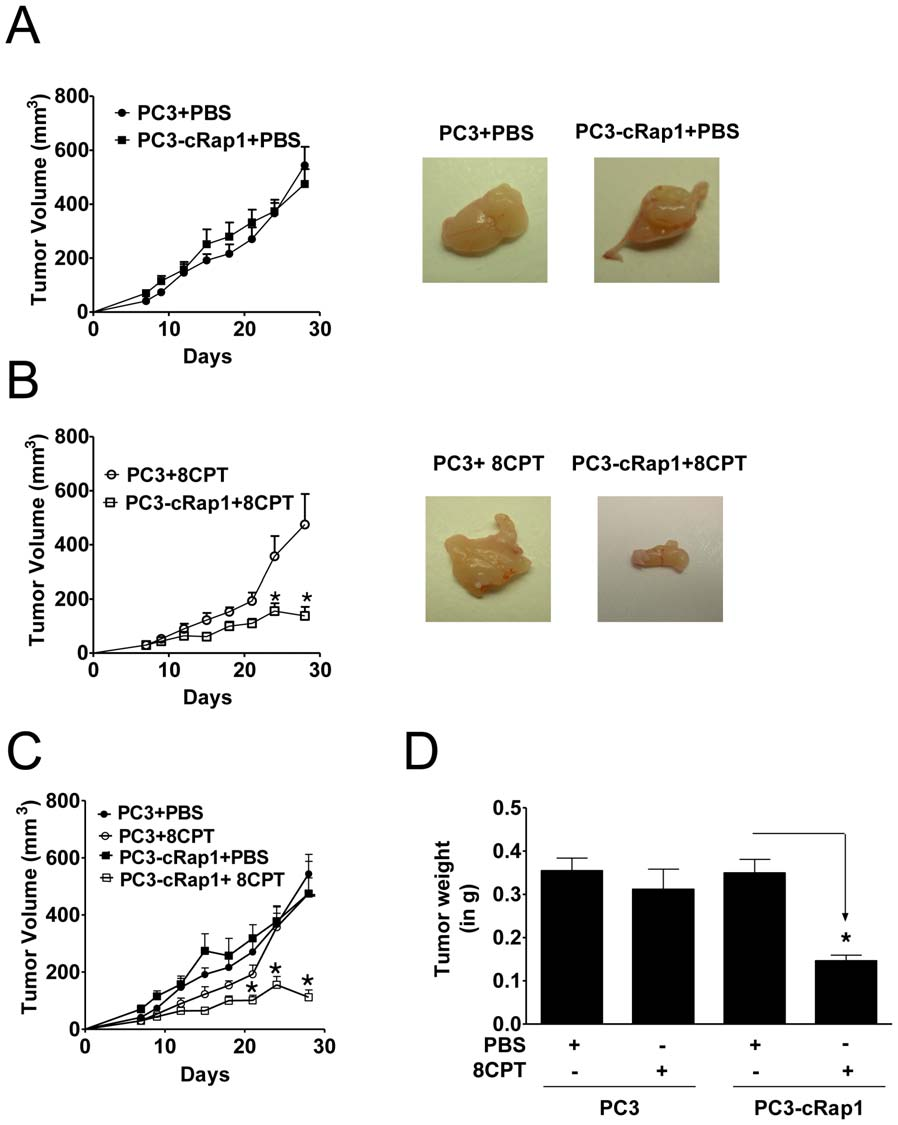
Effect of constitutive Rap1 expression (cRap1) and/or 8CPT treatment on PC3 xenograft growth. (A and B) *Left Panel*: Tumor volume (expressed in mm^3^) of human PC3 and PC3-cRap1 xenografts treated as indicated was measured as described in Methods. Day 0 represents the first day of treatment; *p*<*0.05, n = 7 for each group. *Right Panel*: Images of a representative tumor from each treatment group at the end of the experiment (Day 28). (C) Tumor volume (expressed in mm^3^) of human PC3 and PC3-cRap1 xenografts treated as indicated was measured as in A; *p*<*0.05, n = 7 for each group. (D) Tumors from panel C were weighed at the end of the experiment (Day 28) and expressed in grams; *p<0.05; (*n* = 7).

## Materials and Methods

### Prostate Cancer Cell Lines, Treatments, Antibodies, Plasmids, and Reagents

Epac activator 8-(4-chlorophenylthio)-2-O-methyl-cAMP (8CPT) and PKA activator N6-Benzoyladenosine- 3′, 5′- cyclic monophosphate (6-Bz-cAMP) were purchased from BIOLOG Life Sciences Institute (Bremen, Germany). LNCaP cells and PC3 cells, were obtained from American Tissue Culture Collection (Manassas, Virginia), maintained at 37°C in a humidified atmosphere of 5% CO_2_ in Mediatech RPMI 1640 medium with 10% fetal bovine serum, 50 µg/mL penicillin and 50 U/mL streptomycin. All media and growth reagents were purchased from Gibco BRL (Grand Island, NY). Cells were incubated for 24 h with serum-free media prior to each treatment. Cells were treated overnight with 10 µM 8CPT or 10 µM 6Bz-cAMP dissolved in PBS or PBS alone as a control medium. Cells were further treated for 6 h (unless otherwise stated) with 10 µM CoCl_2_ to mimic hypoxic conditions or with SDF-1α at 200 ng/ml for 20 min. Cells were pretreated with the PKA inhibitors H-89 (10 µM) for 30 minutes before CoCl_2_ treatment. Antibodies to Epac and tubulin were obtained from Santa Cruz Biotechnology (Santa Cruz, CA). Mouse anti–human CD31 and rabbit anti-human VEGF were purchased from Abcam (Cambridge, MA). SDF-1α was obtained from R&D systems (Minneapolis, MN) and H-89 was purchased from Sigma-Aldrich (St Louis, MO), myristoylated PKI was from Invitrogen (Grand Island, NY), and antibodies to VASP or phosphoVASP were from Cell Signaling (Boston, MA).

**Figure 2:**
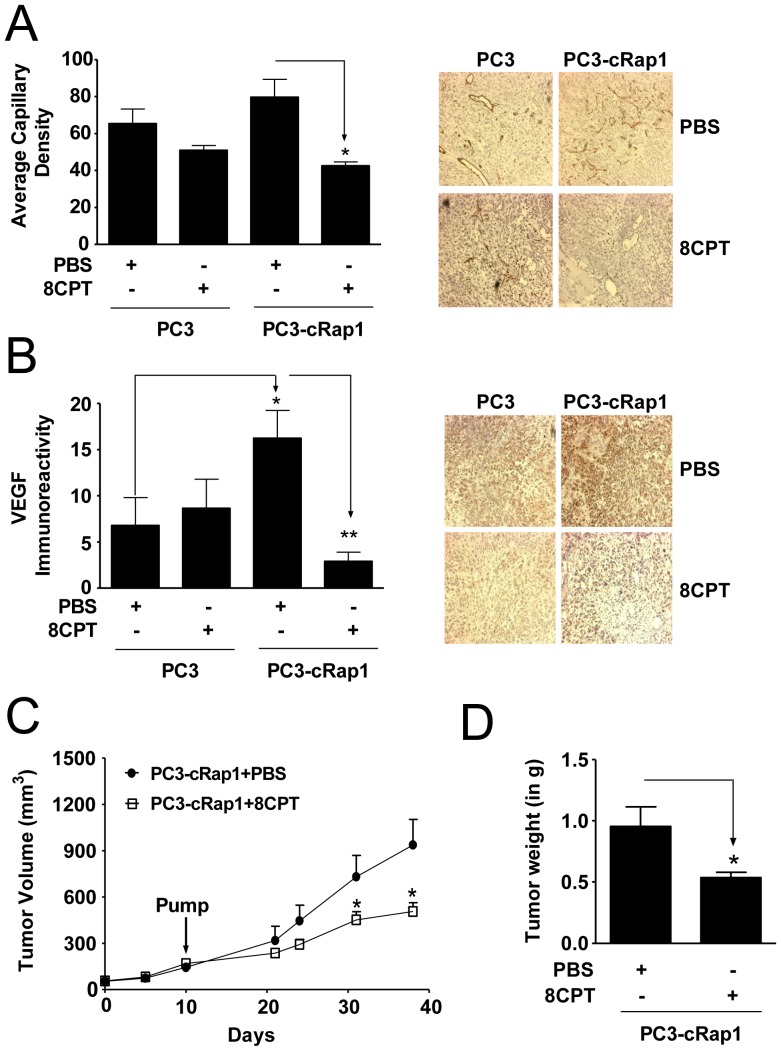
Effect of 8CPT treatment on angiogenesis and tumor growth in PC3 and PC3-cRap1 xenografts. (A and B) CD31 and VEGF immunoreactivity in PC3 and PC3-cRap1 tumors. Immunostaining was measured as described in Methods. *Right Panels*: Representative photomicrographs. *Left Panels*: Quantification of immunohistochemical staining of tumor slices; *p<0.05; n = 7; a.u. = arbitrary units. (C) Tumor volume (expressed in mm^3^) of human PC3-cRap1 xenografts. PC3-cRap1 cells were injected as xenografts at Day 0 and allowed to grow for 10 days before a osmotic minipump containing the treatments was implanted for constant infusion (Day 10, pump). Tumors treated as indicated were measured as described in Methods; *p<0.05; n = 8 for each group. (D) Tumor weight of human PC3-cRap1 xenografts. Tumors from panel C were weighed at the end of the experiment (Day 38) and expressed in grams; *p<0.05; (*n* = 8).

### Ethics Statement

All procedures involving mice complied with the policies of the University of Chicago Institutional Animal Care and Use Committee (IACUC) and were approved by IACUC under the protocol #1196. Mice were housed in the Vivarium in the Gordon Center for Integrative Science at the University of Chicago in dedicated rooms with ventilated cage with HEPA-filtered air (12-hour light/dark cycle). Mice had full access to water and food. Mice were monitored daily by the research staff. The ability to access food and water, hind limb mobility, normal breathing, normal ambulation, and social behavior were evaluated. If an animal appeared to be ill it was monitored for 24 hours and then anesthetized with isoflurane and sacrificed by gaseous agent followed by cervical dislocation. Animals were also euthanized on the recommendation of the veterinarian or clinical staff. All procedures were performed under ketamine/xylazine anesthesia, and all efforts were made to minimize suffering.

### Xenograft Model of Human Prostate Cancer

Male athymic mice (15–20 g, 4–6 weeks of age, National Cancer Institute, Frederick, MD) were inoculated subcutaneously in the lower left flank with 1 × 10^6^ PC3 (PC3-GFP) or PC3-cRap1 (PC3-cRap1A63E-GFP) prostate cancer cells suspended in 200 µL of PBS. The mice were randomized for treatment with either PBS or 2.5 µmol 8CPT, or 2.5 µmol H-89. For this purpose, mice were anesthetized as described above and osmotic minipumps (Alzet model 2004, Durect Corp, Cupertino, CA) were inserted subcutaneously to infuse either PBS, 8CPT or H-89 for 28 days. At the end of the treatment the animals were euthanized, tumors were removed, weighted and processed for immunohistochemistry. Tumor volume was measured using the formula −1/2 × l × w^2^ every other day, where l = length and w = width.

**Figure 3:**
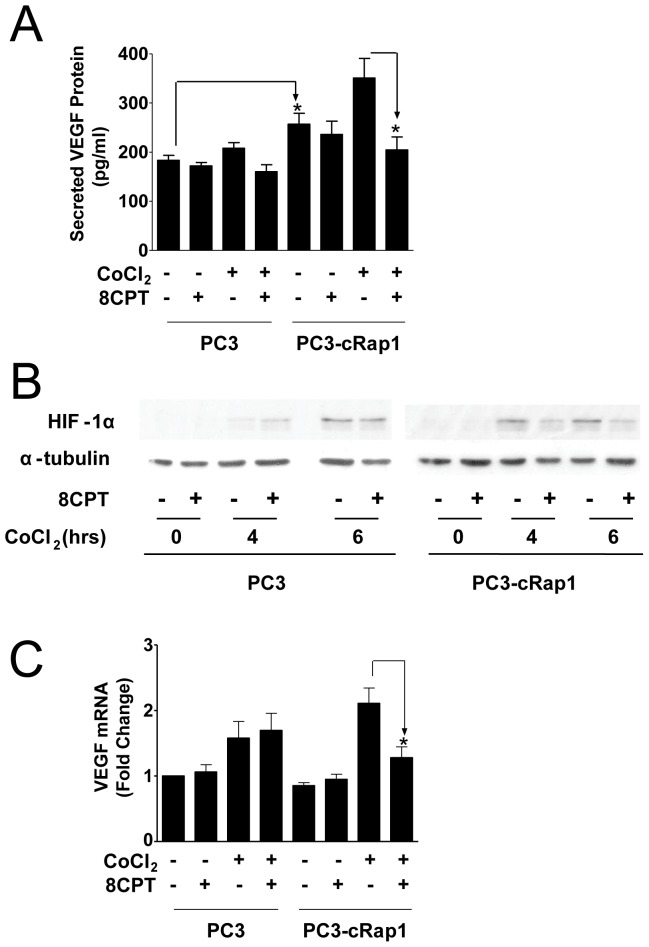
Effect of 8CPT treatment on HIF-1α and VEGF in PC3 and PC3-cRap1 cells under hypoxic-like conditions. (A) VEGF levels were measured by ELISA in PC3 and PC3-cRap1 cells under hypoxic-like conditions (CoCl_2_) as described in Methods; *p<0.05, (n = 3). (B) Immunoblot analysis of nuclear extracts from PC3 and PC3-cRap1 cells treated as indicated. HIF-1α protein levels were determined by immunoblotting with specific antibody; (C) Analysis of VEGF mRNA levels by qPCR in PC3 and PC3-cRap1 cells treated as indicated. Data were normalized to GAPDH levels; *p<0.05 (*n* = 3).

### Immunohistochemistry

Mouse tumors were fixed with 4% paraformaldehyde for 24 h and incubated in 70% ethanol for 48 h prior to paraffin embedding. The embedded tumors were cut into five micron thick sections and stained with hematoxylin and eosin to determine morphology. Sections of tumors infused with 8CPT, H-89 or PBS were immunostained for VEGF, CD31 or Ki67 using the labeled streptavidin-biotin method to measure cell angiogenesis or proliferation. Stained sections were visualized and photographed with a video image analysis system (Scion, Inc., Frederick, MD). An automated cell imaging system was used to quantify VEGF immunohistochemical staining. Capillary density was calculated by counting vessels immunostained with CD31+. At least 6 random fields were counted in a representative section. Values are represented as means plus or minus SEM.

### VEGF ELISA

Cells (5×10^4^) were plated in 24-well plates. When cells were 70–80% confluent, they were treated as described above. Medium was collected from the wells and VEGF levels were determined as per manufacturer’s instructions for Human VEGF ELISA Development kits (PeproTech, Rocky Hill, NJ).

### RNA Isolation and Reverse Transcription/Real-time Polymerase Chain Reaction

RNA was isolated from the cells using QIAshredder and RNeasy^R^ Minikit from Qiagen Biosciences and was subjected to RT-PCR using high capacity cDNA reverse-transcriptase kit (Applied Biosystems, Carlsbad, CA). Quantitative real-time polymerase chain reaction (qPCR) was carried out using Gene Expression Assays from Applied Biosystems and 2X master mix from Applied Biosystems or ABGene on an Applied Biosystems 7300 in a StepOne Plus Real-Time PCR System. The results were quantified as Ct values, where Ct is defined as the threshold cycle of PCR at which amplified product is first detected, and defined as relative gene expression (the ratio of target/control). All reactions were performed in triplicate and normalized to GAPDH (Applied Biosystems, Carlsabad, CA).

### Preparation of Nuclear Extracts and Western Blotting

Sub-confluent PC3 and PC3-cRap1 cells were lysed in cold nuclear buffer (10 mM HEPES, pH 7.9, 1.5 mM MgCl2, 0.5 mM DTT, and 5% glycerol), containing complete protease inhibitors (EMD Chemicals, Paulsboro, NJ). The preparation was centrifuged at 10,000 g for 15 min. The pellet was resuspended in 50 µl of high salt buffer (10 mM HEPES, pH 7.9, 400 mM NaCl, 0.1 mM EDTA, 0.5 mM DTT, and 5% glycerol) and incubated on ice for 20 min. After centrifugation at 14,000 g for 20 min, the supernatant was collected and taken as the nuclear fraction and stored at −80°C. Nuclear proteins (50 µg/well) from control and 8CPT treated cells under hypoxia-like conditions were separated by 7.5% SDS-polyacrylamide gel electrophoresis and transferred to nitrocellulose membranes (Amersham Pharmacia, Piscataway, NJ). The membranes were incubated with primary antibody overnight (HIF-1α, 1∶500; Novus Biologicals, Inc., Littleton, CO) followed by 1 h incubation with secondary anti-mouse antibody, coupled to horseradish peroxidase (1∶2500; Sigma, St. Louis, MO). Chemiluminescent reagents were used to visualize the immunoreactive bands. α-tubulin (1∶3000; Santa Cruz Biotechnology, Santa Cruz, CA) served as the loading control.

### siRNA Transfection

Epac siRNAs (ON-TARGETPlus siRNA, Dharmacon) at 100 nM were transfected into PC3 cells by electroporation (223 v for 23 msec). Cells were analysed 48 hrs after transfection.

### Rap1 Activity Assay

Rap1-GTP levels were measured using a Rap1 Activation Assay Kit (Cell Biolabs, San Diego, CA) following the manufacturer's protocol.

### Statistical Analysis

Data were analyzed using GraphPad Prism v5 (GraphPad Software, Inc.). Data are given as mean ± SE and compared by t-test, Wilcoxon test and one- or two-way ANOVA, as appropriate, followed by the relevant post-hoc t-test to determine p-values. A p-value of <0.05 was considered significant.

**Figure 4:**
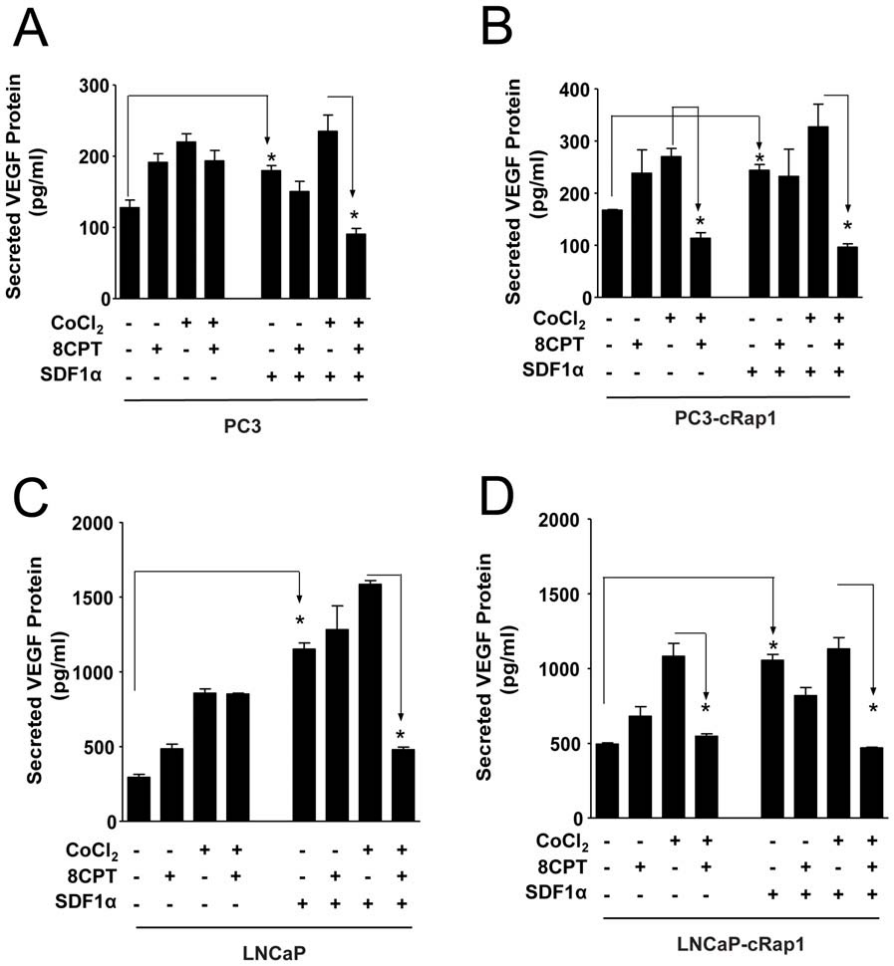
Effect of SDF-1α on secreted VEGF in prostate cancer cells under hypoxic-like conditions. Cells were treated as indicated and VEGF levels were measured by ELISA as described in Methods. (A and B) PC3 or PC3-cRap1 cells; *p<0.05, n = 3. (C and D) LNCaP or LNCaP-cRap1 cells; *p<0.05, n = 2.

**Figure 5:**
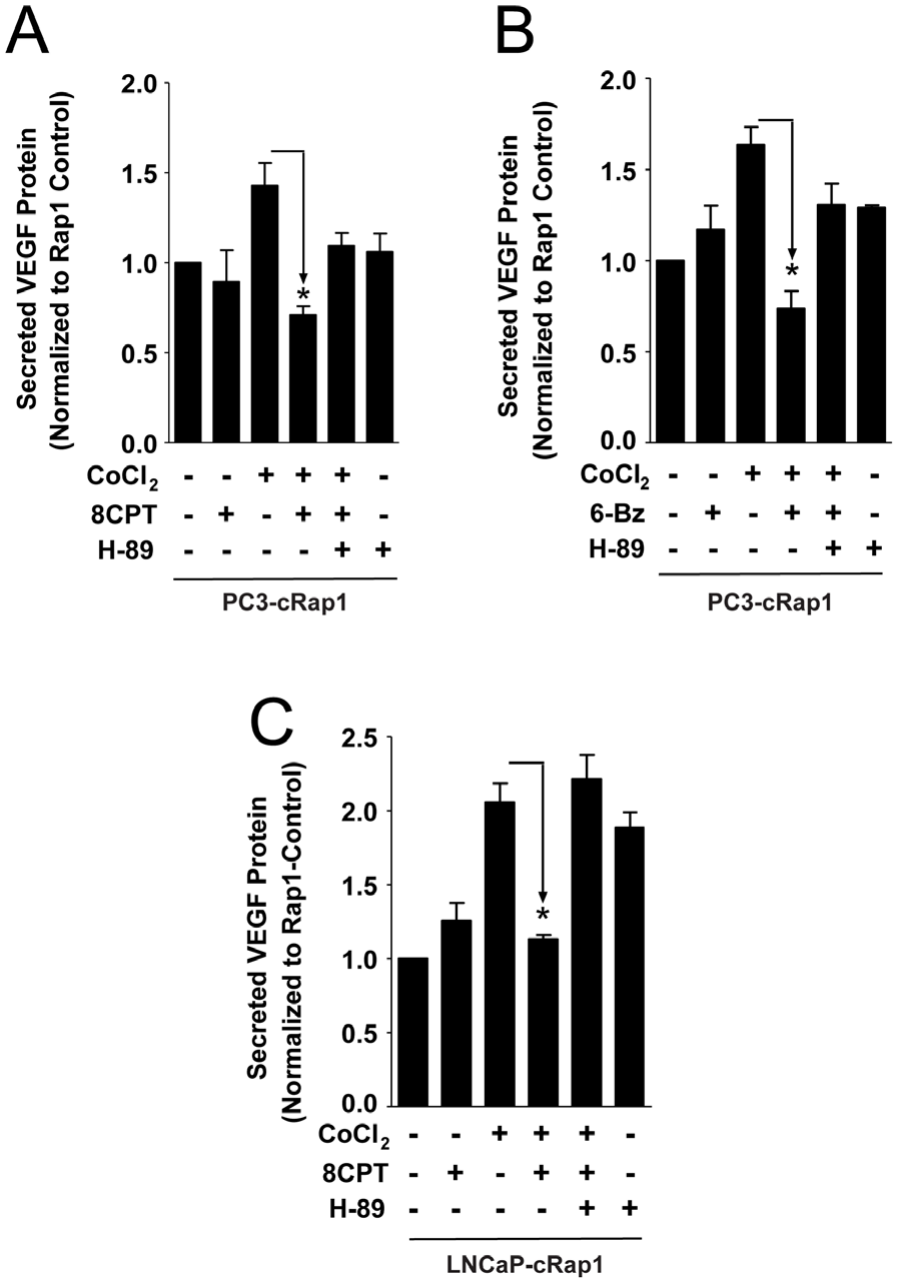
Effect of PKA inhibitor and PKA activator on secreted VEGF in PC3-cRap1 and LNCaP-Rap1 cells under hypoxic-like conditions. Cells were treated as indicated, and VEGF levels were measured by ELISA as described in Methods. (A) Reversal of 8CPT effects by H-89 in PC3-cRap1 cells; *p<0.05, n = 3. (B) Reversal of 6BzcAMP (6Bn) effects by H-89 in PC3-cRap1 cells; *p<0.05, n = 3. (C) Reversal of 8CPT effects by H-89 in PC3-cRap1cells; *p<0.05, n = 3.

## Results

### 8CPT Inhibits Xenograft Growth of Prostate Cells Expressing Activated Rap1

To determine the effect of activated Rap1 on prostate tumor growth, we injected athymic mice with PC3 cells expressing constitutively activated Rap1A63E (PC3-cRap1) or treated with the cAMP derivative 8CPT. For 8CPT delivery, the animals were treated subcutaneously with either PBS or 8CPT using an osmotic mini-pump. 8CPT was administered at a dose of 2.5 µmol over 28 days, based on a previous pilot study showing that this infusion rate was well tolerated by the mice. During the infusion period, the animals maintained normal food and water consumption and showed no evidence of reduced motor function. No gross pathologic abnormalities were observed in major organs, indicating a lack of toxic effects at the 8CPT dose given. The first day of infusion was designated as Day 0, and the animals were sacrificed after 28 days of treatment.

Neither constitutively expressed Rap1 (PC3-cRap1+PBS) nor 8CPT treatment alone (PC3+8CPT) significantly altered tumor growth in PC3 xenografts (Fig. 1A, 1C). However, 8CPT dramatically reduced tumor growth in PC3-cRap1 xenografts (Fig. 1B, 1C). Analysis of tumor weights confirmed a >50% decrease in tumors from PC3-cRap1 mice treated with 8CPT (Fig. 1D). Both activated Rap1 and 8CPT treatment were required for this effect, since no change in tumors was observed in 8CPT-treated PC3 xenografts or PBS-treated Rap1 xenografts.

**Figure 6:**
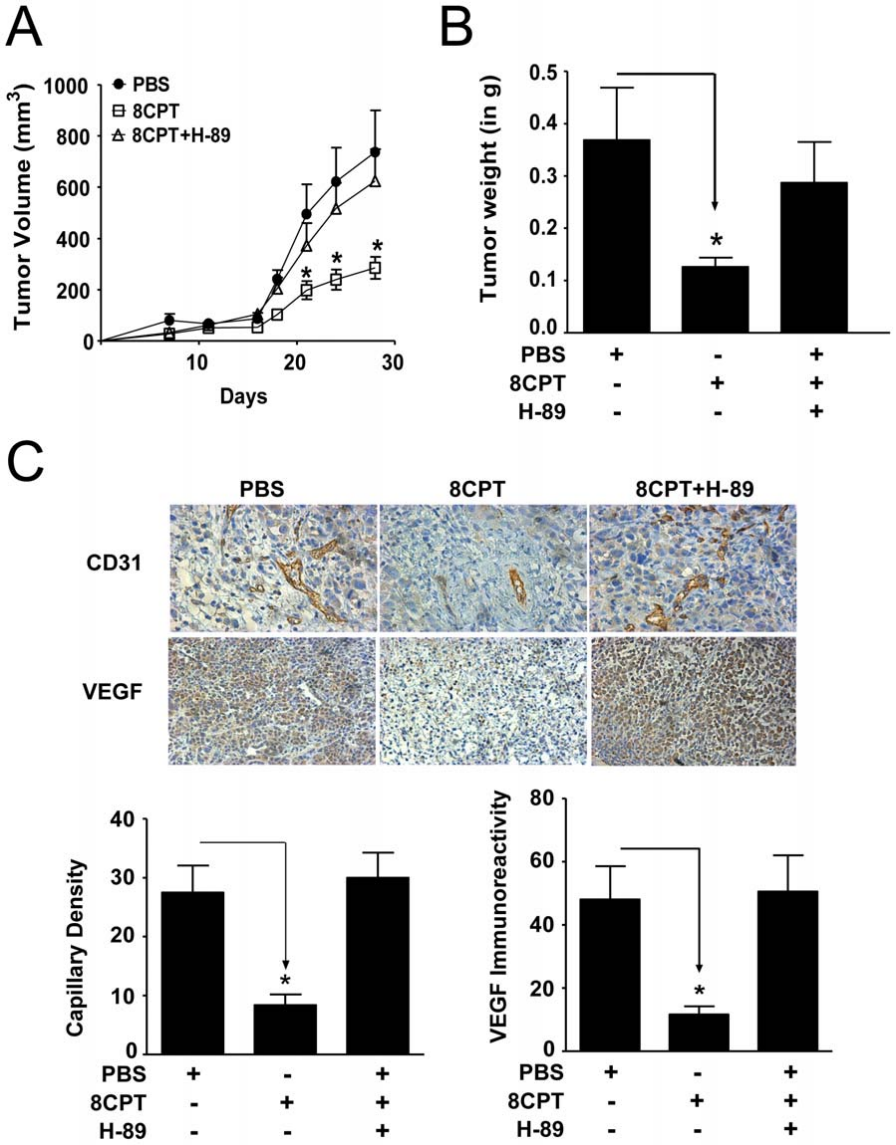
Effect of PKA inhibitor on tumor growth and angiogenesis in PC3-cRap1 xenografts. (A and B) The PKA inhibitor, H-89 (2.5 µmol), reversed tumor growth inhibition and tumor weight reduction mediated by 8CPT in PC3-cRap1 xenografts. PC3-cRap1 tumors from mice infused with PBS, 8CPT, or H-89 were measured and tumor volume determined as described in Methods; * p<0.05; n = 8 for each treatment group. (C) CD31 and VEGF immunoreactivity in PC3-cRap1 tumors treated as indicated. Immunostaining was measured as described in Methods. *Upper Panels*: Representative photomicrographs. *Lower Panels*: Quantification of immunohistochemical staining of tumor slices; *p<0.05; n = 8 for each treatment group; a.u. = arbitrary units.

**Figure 7:**
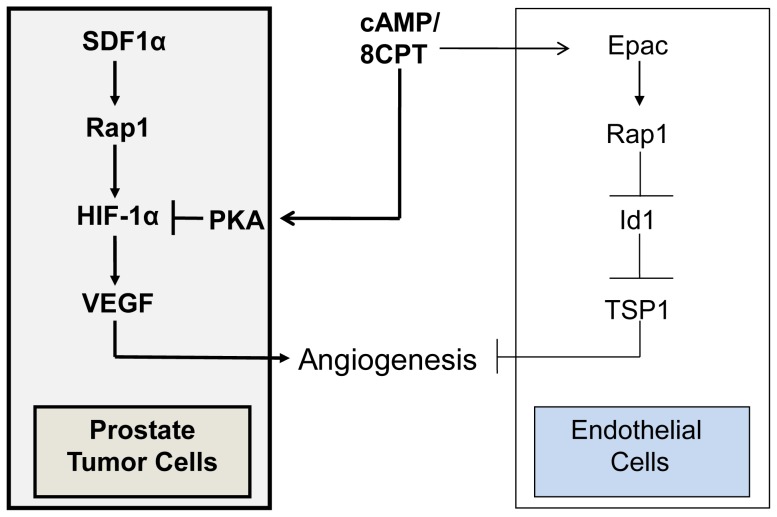
Schematic depicting interplay between Rap1 and PKA in the tumor microenvironment.

### 8CPT Inhibits Angiogenesis in PC3-cRap1 Xenografts

To monitor the effect of 8CPT on angiogenesis in the prostate tumors, formalin-fixed tumor slices were immunostained with antibodies to CD31 and VEGF, an endothelial cell marker and a proangiogenic factor, respectively. Intra-tumor blood vessels (CD31+) were identified by vessel morphology and counted (Fig. 2A). Tumors from the PC3 group of animals infused with PBS or 8CPT had similar numbers of blood vessels and levels of VEGF immunoreactivity (Fig. 2A, 2B). The overall VEGF immunoreactivity in the PC3-cRap1 mouse tumors was increased relative to the PC3 mouse tumors without significantly affecting capillary density. Surprisingly, treatment with 8CPT, when compared to PBS, dramatically reduced both the vessel density and the VEGF immunoreactivity in PC3-cRap1 tumors. These results indicate that constitutively active Rap1 induces VEGF production, and 8CPT antagonizes this effect. Furthermore, 8CPT reduces blood vessel formation, which may contribute to the inhibition of prostate tumor growth in these mice.

### 8CPT Reduces Continued Growth of Pre-formed Prostate Tumors Expressing Rap1

In our initial experiments (Fig. 1), we injected PC3-cRap1 cells and treated with 8CPT on the same day (Day 0) to prevent tumor growth. To determine whether 8CPT could also block ongoing tumor cell growth, we injected PC3-cRap1 cells into mice and allowed them to grow into a palpable tumor before treatment with 8CPT via osmotic mini pumps (Fig. 2C). Similar to the previous regimen, 8CPT treatment of pre-existing tumors caused more than 43% reduction in tumor weight when compared to PBS infused animals (Fig. 2D). Analysis of tumor cell proliferation by immunostaining with Ki67 antibody showed only a modest decrease following 8CPT treatment, suggesting that the reduction in cell number is also due to cell loss (Fig. S1). However, apoptosis alone is unlikely to be responsible, since TUNEL staining did not show a significant difference (data not shown). The results indicate that 8CPT not only acts to prevent tumor growth but also reduces continued growth of pre-formed tumors.

### 8CPT Inhibits Angiogenic Inducers in PC3-cRap1 Cells Under Hypoxic Conditions

To understand the mechanism by which 8CPT suppresses VEGF in prostate tumor cRap1 xenografts, we developed cell culture conditions to mimic this effect. Solid tumor cells often grow under hypoxic conditions, and hypoxia promotes angiogenesis. Therefore, we analyzed VEGF protein levels in media isolated from cells pretreated overnight with 8CPT in reduced serum and then further treated with CoCl_2_ for 6 hours to generate hypoxia-like conditions. In PC3-cRap1 cells, 8CPT treatment decreased VEGF production only under hypoxic-like conditions (CoCl_2_) (Fig. 3A). By contrast, there was no marked reduction in VEGF production in PC3 cells following 8CPT treatment with concurrent exposure to CoCl_2_ (Fig. 3A). These results suggest that the 8CPT-induced inhibition of VEGF expression observed in PC3-cRap1 tumors can be recapitulated *in vitro* by mimicking a hypoxic environment.

HIF-1α is a heterodimeric transcription factor that is upregulated in hypoxic tumor cells and promotes angiogenesis by enabling transcription of pro-angiogenic genes like VEGF [30]. To determine whether HIF-1α inhibition could be the mediator of the 8CPT effect on VEGF levels, we analyzed HIF-1α protein expression in nuclear extracts from PC3 and PC3-cRap1 cells treated with 8CPT and CoCl_2_. As expected, a significant increase in the level of HIF-1α protein was seen both in the PC3 and PC3-cRap1 cells following CoCl_2_ treatment. However, HIF-1α protein levels decreased when PC3-cRap1 cells but not PC3 cells were pretreated with 8CPT (Fig. 3B). Consistent with this result, we observed a significant decrease in VEGF mRNA only in PC3-cRap1 cells treated with both CoCl_2_ and 8CPT (Fig. 3C).

### 8CPT Regulates VEGF Production in Prostate Cell Lines Treated with a Physiologic Activator of Rap1

Rap1 is activated by a number of endogenous factors including Stromal Derived Factor 1α (SDF-1α), a chemokine that binds to the CXCR4 receptor and facilitates homing of bone marrow-derived cells [31], [32]. As shown previously [31], treatment of PC3 cells with SDF-1α for 20 minutes markedly increased the level of activated endogenous Rap1-GTP (Fig. S2). We did not observe elevated levels of endogenous Rap1-GTP in either PC3 or PC3-cRap1 cells following overnight treatment of cells with 8CPT. These results indicate that either acute SDF-1α treatment or constitutive Rap1 activation but not sustained 8CPT treatment leads to elevated Rap1 activity in prostate tumor cells.

To determine whether Rap1 activated in response to SDF-1α acts similarly to constitutively activated Rap1, we investigated the effect of SDF-1α and 8CPT on secreted VEGF. SDF-1α induced VEGF production in PC3 cells when compared to untreated PC3 cells (Fig. 4A), and this induction was blocked in PC3 cells expressing Rap1GAP, a Rap1 GTPase that inactivates Rap1 (Fig. S3). Additionally, treatment with CoCl_2_ induced further VEGF production in SDF-1α-stimulated PC3 cells, similar to CoCl_2_ treatment of PC3-cRap1 cells (Fig. 4A, 4B). As with PC3-cRap1 cells, 8CPT reversed the induction of VEGF production in SDF-1α-treated PC3 cells under hypoxic conditions.

Similar results were also observed in a less aggressive, androgen-dependent prostate tumor cell line, LNCaP, that either was treated with SDF-1α or with stably expressed constitutive Rap1 (Fig. 4C, 4D). These results indicate that the regulation of VEGF production by 8CPT under hypoxic conditions is not limited to one cell type and is observed in response to physiological as well as constitutive activation of Rap1.

### 8CPT Regulates VEGF Production Downstream of Rap1

To understand the role of Rap1 activation in the regulation of VEGF production, similar studies involving 8CPT or CoCl_2_ treatment were conducted in unstimulated or SDF-1α-stimulated PC3 cells that stably express Rap1GAP. Overall, the VEGF levels were reduced as a result of Rap1GAP expression (Fig. S4). Although induction due to SDF-1α was entirely suppressed, CoCl_2_ still stimulated VEGF production in the presence of Rap1GAP, consistent with HIF-1α stabilization. Furthermore, overnight pretreatment with 8CPT still reduced CoCl_2_-stimulated VEGF production, consistent with our observation that 8CPT acts upstream of HIF-1α to reduce it expression levels (see Fig. 3B). Finally, 8CPT pretreatment did not reduce constitutive Rap1 activity (Fig. S2), suggesting that it acts downstream of Rap1. Taken together, the data suggest that 8CPT inhibits VEGF protein production by inhibiting Rap1 or hypoxic induction of HIF-1α.

### 8CPT Regulates VEGF Production via PKA in Prostate Tumor Cells

Although 8CPT activates Rap1 via Epac in endothelial cells [8], our present results indicate that 8CPT antagonizes activated Rap1 function in epithelial prostate tumor cells suggesting that a different mechanism is responsible. Consistent with this hypothesis, partial depletion of Epac1 and Epac2 by siRNAs did not have any effect on secreted VEGF in PC3-cRap1 cells (Fig. S5) although we cannot rule out a role for Epac.

8CPT can activate PKA although its affinity for Epac is 107-fold greater [19], [33]. To test this possibility, we determined whether 8CPT stimulates PKA in PC3 and PC3-cRap1 cells using VASP phosphorylation as an indicator of PKA activation. 8CPT activated PKA similar to 6-Benzoyl-cAMP (6BzcAMP), a more selective PKA activator (Fig. S6). We then examined the ability of two PKA inhibitors, H-89 or PKI, to reverse the inhibition of VEGF production mediated by 8CPT. Treatment of PC3-cRap1 cells exposed to CoCl_2_ and 8CPT with the PKA inhibitors partially rescued the inhibition of VEGF production in PC3-cRap1 cells (Fig. 5A; Fig. S7). Furthermore, 6BzcAMP mimicked 8CPT by inhibiting VEGF production in PC3-cRap1 cells under hypoxic conditions, and this inhibition was partially reversed by H-89 or PKI (Fig. 5B; Fig. S8). Taken together, these results suggest that 8CPT acts as an inhibitor of pro-angiogenic factors in hypoxic Rap1-stimulated prostate tumor cells via PKA activation.

To test this mechanism in prostate tumors, we determined whether PKA inhibition could reverse the tumor growth and angiogenesis inhibition mediated by 8CPT *in vivo*. Following injection of athymic mice with actively proliferating PC3-cRap1 cells, the animals were treated subcutaneously with PBS, 8CPT, or H-89+8CPT using osmotic mini-pumps. As observed previously in mice injected with PC3-cRap1 cells, the tumors increased in size over time but tumor growth was significantly reduced upon treatment with 8CPT (Fig. 6A). However, simultaneous treatment with 8CPT and H-89 reversed the tumor growth inhibition caused by 8CPT in PC3-cRap1 xenografts. Similarly, the 65% decrease in tumor weight of 8CPT-treated animals was rescued by concurrent treatment with H-89 (Fig. 6B). Finally, as shown in Fig. 6C, immunohistochemical analysis of CD31 and VEGF protein expression in sections from mice infused with PBS, 8CPT, or H-89+8CPT indicated that the PKA inhibitor H-89 also reversed the reduction in CD31 and VEGF immunoreactivity mediated by 8CPT in the PC3-cRap1 xenografts. Taken together, these results indicate that PKA inhibition could reverse the tumor growth inhibition and angiogenesis inhibition mediated by 8CPT *in vivo*.

## Discussion

Utilizing a combination of analyses from both *in vitro* and *in vivo* systems, our study provides evidence that a novel interplay between Rap1, Epac, and PKA regulates tumor-microenvironment induction of angiogenesis. We showed previously that sustained activation of Rap1 by 8CPT/Epac or cRap1 inhibits endothelial cell chemotaxis and angiogenesis [8], [9]. To test the importance of Rap1 activation in a prostate tumor xenograft model, we analyzed the effects of 8CPT or cRap1 on PC3 cells. 8CPT had no effect on tumor growth in parental PC3 cells but significantly reduced not only tumor size but also VEGF and angiogenesis in animals injected with PC3 cells expressing cRap1. Surprisingly, 8CPT inhibited VEGF induction and prostate tumor growth by a mechanism involving PKA rather than Epac/Rap1 activation. Our results indicate that Rap1 activation in prostate tumor cells promotes angiogenesis under hypoxic-like conditions via HIF-1α and VEGF, and PKA activation antagonizes this induction (see scheme depicted in Fig. 7). In addition, our studies suggest that endothelial cells preferentially activate Epac in response to sustained 8CPT treatment whereas prostate epithelial cells preferentially activate PKA.

Both Rap1 and PKA have been linked previously to cancer in a prostate epithelial cell model. Androgen-dependent and -independent prostate cancer cell lines express endogenous Rap1 [31], [34]. Rap1 is activated by a variety of growth factors including platelet-derived growth factor (PDGF), epidermal growth factor (EGF), endothelin, and lysophosphatidic acid (LPA) through different mechanisms including cAMP activation of Epac [35]. Treatment of LNCaP but not PC3 prostate cancer cells with forskolin or cAMP causes Rap1 phosphorylation and activation by protein kinase A (PKA) [36], and activated Rap1 has been shown to induce the MAPK pathway by stimulating B-Raf [37], [38]. However, the results we describe here suggest that a different mechanism is responsible for the observed effects. First, we see the effects in both LNCaP and PC3 cells. Furthermore, rather than potentiating Rap1 activity, PKA acts in an antagonistic fashion to suppress Rap1-induced VEGF production.

Rap1 has been implicated in the progression of tumorigenesis largely through regulation of cell adhesion and cell-cell junctions. For example, Rap1 promotes invasion and metastasis in PC3 cells via regulation of the integrins α4β3 and αvβ3 [31]. By contrast, a recent study reported that 8CPT inhibits cell migration in prostate cancer cells via activation of Epac [39]. Our results suggest that this effect could be due in part to PKA activation by 8CPT, an interpretation consistent with the observed antagonism between Rap1 and PKA in prostate tumor cells. Our results also suggest that activated Rap1 in prostate tumor cells sensitizes the cells to inhibition of HIF-1α activity by PKA. Whether this mechanism involves signaling through an integrin receptor or another mechanism remains to be determined.

A recent study based on Rap1 knockout mouse models reported that Rap1b induces angiogenesis in endothelial cells via VEGFR2 activation by an integrin-dependent mechanism [40]. This result is consistent with regulation of cell adhesion [41] and cell-cell junctions [42] by Rap1 during development. The results we reported previously using human endothelial xenografts [8] suggest that nonphysiological stimulation of Rap1 in endothelial cells by expression of a constitutively activated Rap1 mutant or Epac activation by prolonged 8CPT treatment can actually suppress angiogenesis. This model could be relevant to the tumor microenvironment since elevated SDF-1α levels can lead to constitutive Rap1 activation. In the present study we show that Rap1 in epithelial prostate tumor cells promotes VEGF production and is therefore pro-angiogenic. However, in this case, 8CPT again appears to inhibit angiogenesis by a mechanism involving suppression of VEGF production in the tumor cells by PKA. Thus, drug treatments involving the cAMP pathway can coordinately inhibit angiogenesis via both epithelial tumor and endothelial cell types.

Our data suggests that the activation of PKA in Rap1-activated prostate tumor cells can prevent or block tumor growth. Our results show an anti-angiogenic role for PKA in prostate tumor cells and these results complement a previous study showing that PKA also suppresses smooth muscle [43] and blood vessel growth [24]. The effect of 8CPT is consistent with those of other anti-angiogenic inhibitors that do not kill tumor cells but act to prevent growth. Treatment of cells with 8CPT in combination with chemotherapeutic agents or other toxic drugs could be a potent treatment regimen. To avoid potential complications from this approach, future studies could be directed at more specific targeting of 8CPT or the identification of drugs that mimic its effects by targeting selected downstream effectors of PKA.

## Supporting Information

Figure S1 **Ki67 staining of PC3-Rap1 tumors treated with control buffer (PBS) or 8CPT.** Tumors from Fig. 1 were isolated, fixed, and stained for immunohistochemistry using an anti-Ki67 antibody. Positive cells were counted in 6 random fields and the average number of positive cells/field plotted.
(PDF)

Figure S2 **Effects of SDF-1α and 8CPT on Rap1 activation.** Rap1 activation was assayed by a Rap1-GTP assay. Either PC3 or PC3-cRap1 cells were treated with PBS (Con), SDF-1α (200 ng/ml for 20 minutes) or 8CPT overnight. Rap1-GTP was isolated by pulldown assay as described in Methods. GTPγS was added as a positive control for Rap1-GTP (GTPγS) formation as indicated.
(PDF)

Figure S3 **Effect of Rap1GAP on VEGF secretion in PC3 cells.** PC3 cells were stably transfected with Rap1GAP as previously described (7) and then assayed for VEGF protein levels by ELISA as described in Methods.
(PDF)

Figure S4 **Effect of Rap1GAP on VEGF secretion in PC3 cells treated with 8CPT, SDF-1α or hypoxic-like conditions.** PC3 cells were stably transfected with Rap1GAP as in Figure S3. Cells were then treated with 8CPT, SDF-1α or CoCl_2_ as described in Methods and secreted VEGF protein was measured.
(PDF)

Figure S5 **Effect of siRNA depletion of Epac1 and Epac2 on VEGF secretion in PC3-cRap1 cells.** PC3-cRap1 cells were transfected with control siRNAs (siCon) or siRNAs for Epac1 (siEpac1) and Epac2 (siEpac2) 48 hours prior to treatment with 8CPT and/or CoCl_2_. *Left panel*: secreted VEGF levels were assayed by ELISA in the absence or presence of siRNA for Epac1 and Epac2 (siEpac1+2). *Right panel*: immunoblots of cell lysates resolved by SDS PAGE. Samples were probed with anti-Epac1 or anti-Epac2 antibody or with tubulin.
(PDF)

Figure S6 **Activation of PKA in PC3 and PC3-cRap1 cells.** Cells were treated with PBS (Con), 8CPT or 6BzcAMP (6Bn) overnight and then incubated in the presence or absence of CoCl_2_ as described in Methods. PKA activation was assayed by phosphorylation of VASP. Cell lysates were immunoblotted with anti-phosphoVASP or anti-tubulin antibodies.
(PDF)

Figure S7 **8CPT inhibits secreted VEGF levels and myristoylated PKI reverses it.** PC3-cRap1 cells were untreated or exposed to 8CPT overnight and/or treated with CoCl_2_. Prior to CoCl_2_ administration, cells were pretreated with either buffer or myristoylated PKI as described for H89.
(PDF)

Figure S8 **6BzcAMP inhibits secreted VEGF levels and myristoylated PKI reverses it.** PC3-cRap1 cells were untreated or exposed to 6BzcAMP (6Bn) overnight or treated with CoCl_2_ as described in Methods. Prior to CoCl_2_ administration, cells were pretreated with either buffer or myristoylated PKI.
(PDF)
