# Supplementary figures and images for: A Novel Interplay between Rap1 and PKA Regulates Induction of Angiogenesis in Prostate Cancer
Source: PLoS One. 2012 Nov 15;7(11):e49893. doi: 10.1371/journal.pone.0049893 (PMC3499522; doi:10.1371/journal.pone.0049893)

Figure S1

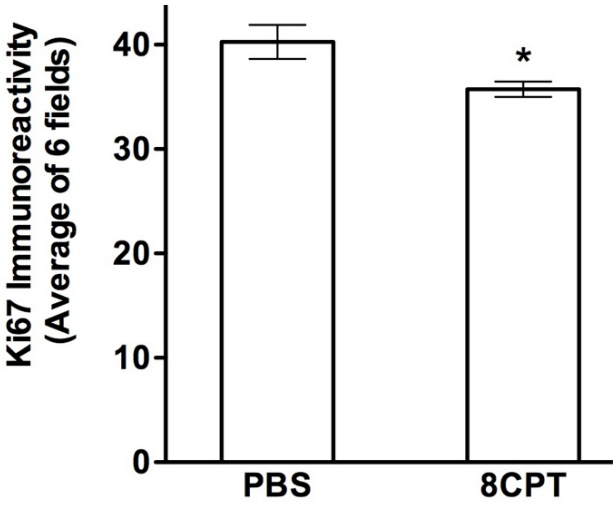

Supplement: Figure S1 — Ki67 staining of PC3-Rap1 tumors treated with control buffer (PBS) or 8CPT. Tumors from Fig. 1 were isolated, fixed, and stained for immunohistochemistry using an anti-Ki67 antibody. Positive cells were counted in 6 random fields and the average number of positive cells/field plotted. (PDF) [file pone.0049893.s001.pdf]

Figure S2

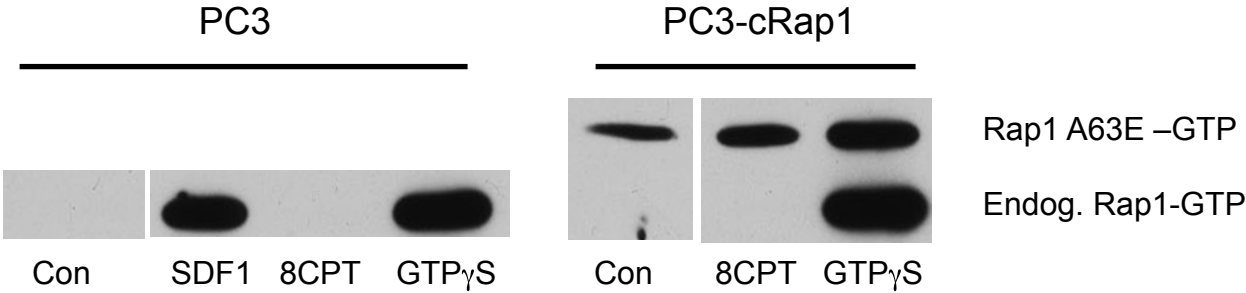

Supplement: Figure S2 — Effects of SDF-1α and 8CPT on Rap1 activation. Rap1 activation was assayed by a Rap1-GTP assay. Either PC3 or PC3-cRap1 cells were treated with PBS (Con), SDF-1α (200 ng/ml for 20 minutes) or 8CPT overnight. Rap1-GTP was isolated by pulldown assay as described in Methods. GTPγS was added as a positive control for Rap1-GTP (GTPγS) formation as indicated. (PDF) [file pone.0049893.s002.pdf]

Figure S3

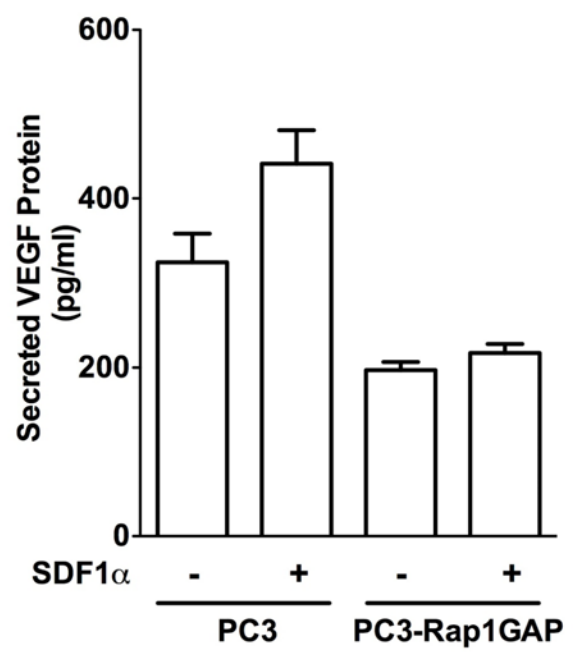

Supplement: Figure S3 — Effect of Rap1GAP on VEGF secretion in PC3 cells. PC3 cells were stably transfected with Rap1GAP as previously described (7) and then assayed for VEGF protein levels by ELISA as described in Methods. (PDF) [file pone.0049893.s003.pdf]

Figure S4

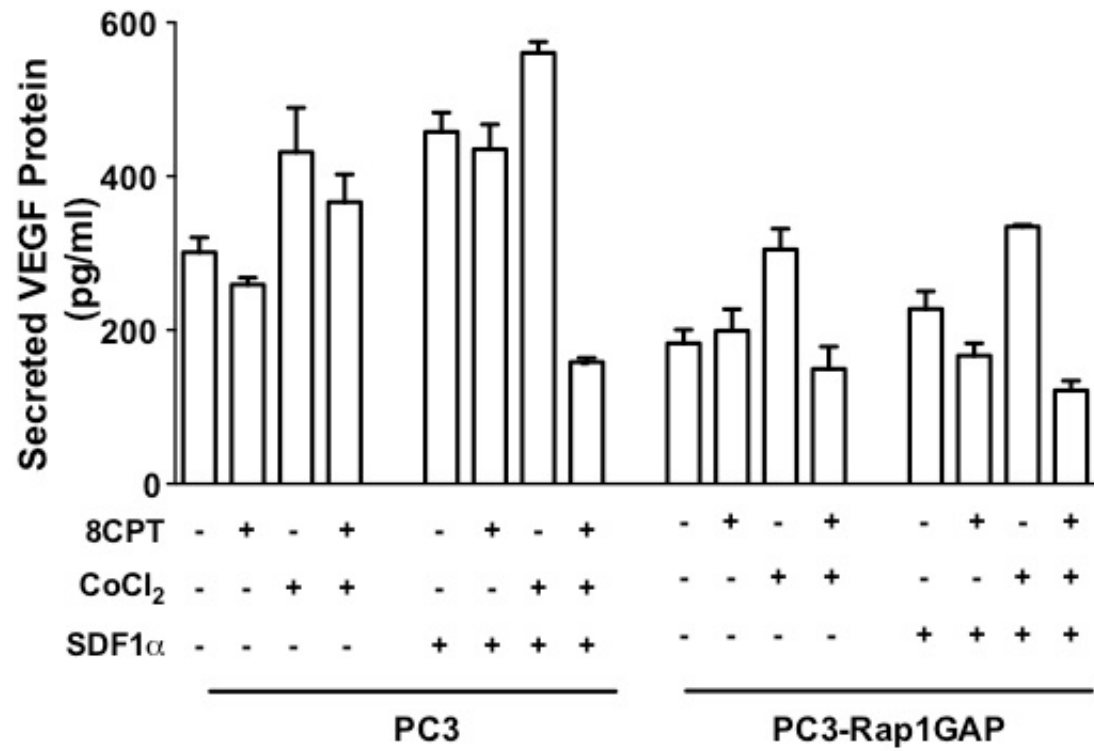

Supplement: Figure S4 — Effect of Rap1GAP on VEGF secretion in PC3 cells treated with 8CPT, SDF-1α or hypoxic-like conditions. PC3 cells were stably transfected with Rap1GAP as in Figure S3. Cells were then treated with 8CPT, SDF-1α or CoCl2 as described in Methods and secreted VEGF protein was measured. (PDF) [file pone.0049893.s004.pdf]

Figure S5

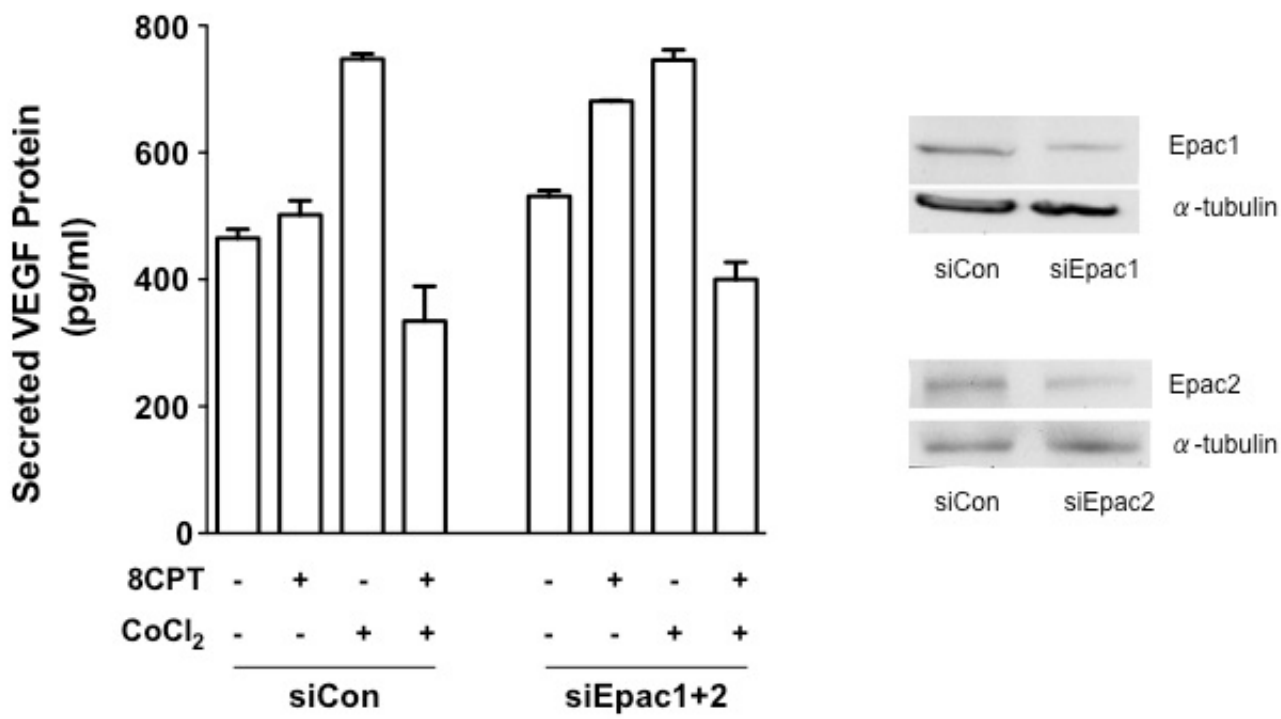

Supplement: Figure S5 — Effect of siRNA depletion of Epac1 and Epac2 on VEGF secretion in PC3-cRap1 cells. PC3-cRap1 cells were transfected with control siRNAs (siCon) or siRNAs for Epac1 (siEpac1) and Epac2 (siEpac2) 48 hours prior to treatment with 8CPT and/or CoCl2. Left panel: secreted VEGF levels were assayed by ELISA in the absence or presence of siRNA for Epac1 and Epac2 (siEpac1+2). Right panel: immunoblots of cell lysates resolved by SDS PAGE. Samples were probed with anti-Epac1 or anti-Epac2 antibody or with tubulin. (PDF) [file pone.0049893.s005.pdf]

Figure S6

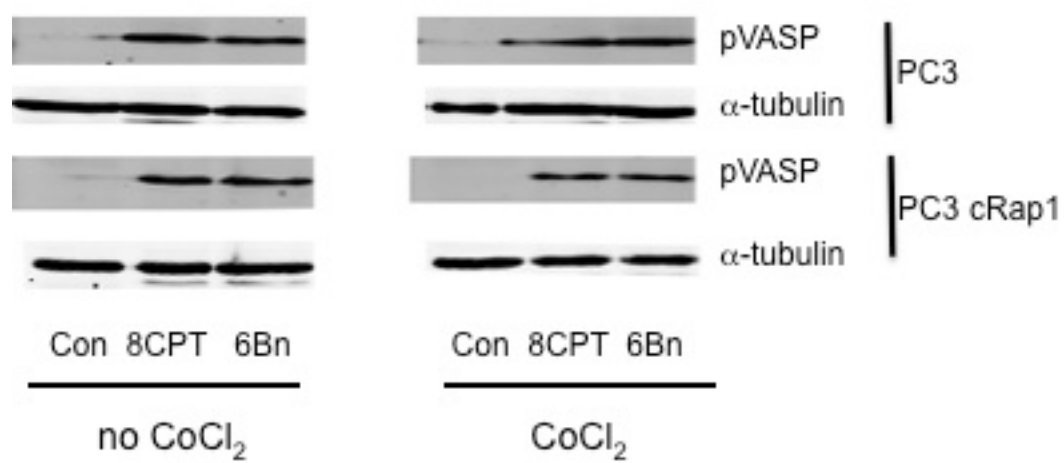

Supplement: Figure S6 — Activation of PKA in PC3 and PC3-cRap1 cells. Cells were treated with PBS (Con), 8CPT or 6BzcAMP (6Bn) overnight and then incubated in the presence or absence of CoCl2 as described in Methods. PKA activation was assayed by phosphorylation of VASP. Cell lysates were immunoblotted with anti-phosphoVASP or anti-tubulin antibodies. (PDF) [file pone.0049893.s006.pdf]

Figure S7

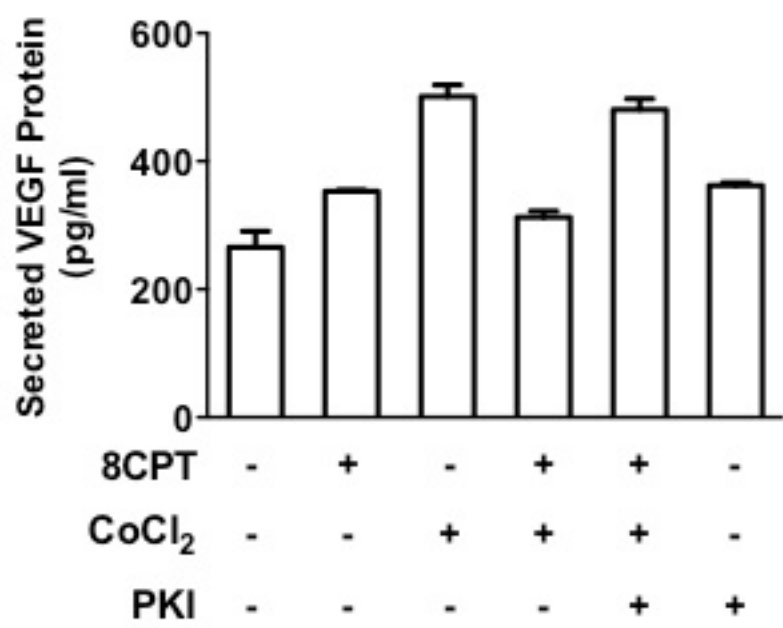

Supplement: Figure S7 — 8CPT inhibits secreted VEGF levels and myristoylated PKI reverses it. PC3-cRap1 cells were untreated or exposed to 8CPT overnight and/or treated with CoCl2. Prior to CoCl2 administration, cells were pretreated with either buffer or myristoylated PKI as described for H89. (PDF) [file pone.0049893.s007.pdf]

Figure S8

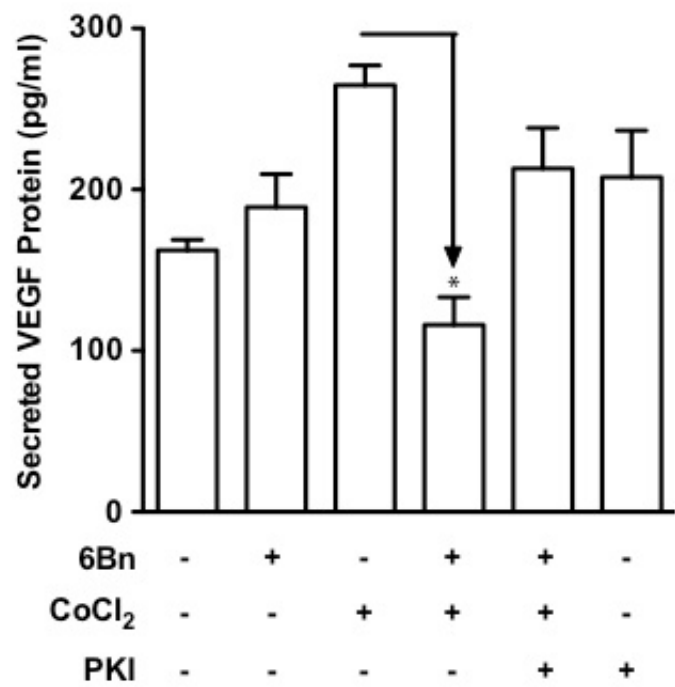

Supplement: Figure S8 — 6BzcAMP inhibits secreted VEGF levels and myristoylated PKI reverses it. PC3-cRap1 cells were untreated or exposed to 6BzcAMP (6Bn) overnight or treated with CoCl2 as described in Methods. Prior to CoCl2 administration, cells were pretreated with either buffer or myristoylated PKI. (PDF) [file pone.0049893.s008.pdf]
